# Coevolutionary Constraints? The Environment Alters Tripartite Interaction Traits in a Legume

**DOI:** 10.1371/journal.pone.0041567

**Published:** 2012-07-30

**Authors:** Katy D. Heath, Katie E. McGhee

**Affiliations:** 1 Department of Plant Biology, University of Illinois at Urbana-Champaign, Urbana, Illinois, United States of America; 2 Department of Animal Biology, University of Illinois at Urbana-Champaign, Urbana, Illinois, United States of America; University of Arkanas, United States of America

## Abstract

Third party species, which interact with one or both partners of a pairwise species interaction, can shift the ecological costs and the evolutionary trajectory of the focal interaction. Shared genes that mediate a host’s interactions with multiple partners have the potential to generate evolutionary constraints, making multi-player interactions critical to our understanding of the evolution of key interaction traits. Using a field quantitative genetics approach, we studied phenotypic and genetic correlations among legume traits for rhizobium and herbivore interactions in two light environments. Shifts in plant biomass allocation mediated negative phenotypic correlations between symbiotic nodule number and herbivory in the field, whereas positive genetic covariances suggested shared genetic pathways between nodulation and herbivory response. Trait variance-covariance (G) matrices were not equal in sun and shade, but nevertheless responses to independent and correlated selection are expected to be similar in both environments. Interactions between plants and aboveground antagonists might alter the evolutionary potential of traits mediating belowground mutualisms (and vice versa). Thus our understanding of legume-rhizobium genetics and coevolution may be incomplete without a grasp of how these networks overlap with other plant interactions.

## Introduction

Though we often study the evolution of species in isolation, organisms in nature engage simultaneously in interactions with multiple species [1]–[3]. The ecological and evolutionary implications of complex, multi-player interactions are only beginning to be explored [4]; however, it has become clear that multi-player interactions have significant potential to alter ecological and evolutionary processes, and that these effects are impossible to estimate by studying pairwise species interactions [1], [4]–[6]. Third-party species, which in combination with a focal pairwise interaction constitute tripartite interactions, have been shown to change the ecological costs or benefits in interactions [7], alter the strength or direction of natural selection [8]–[10], or even categorically shift an interaction from mutualism to parasitism [11]. We cannot fully understand how organisms adapt to such multi-player interactions without studying the genetic architecture of the relevant interaction traits, since these traits are likely underpinned by complex and overlapping genetic response networks and thus might not evolve independently. Such investigations can shed light on variation in coevolutionary interactions and the resulting geographic mosaics [12].

Legume-rhizobium-insect interactions provide a tractable system with which to investigate the evolutionary and ecological effects of tripartite interactions, in part because they can be manipulated in the greenhouse and field [13], [14]. Rhizobia are soil bacteria that, when in symbiosis, are housed in specialized organs called nodules on the roots of legume plants. While in the nodules, rhizobia convert nitrogen to plant-available forms in return for carbon from plant photosynthesis. The interaction is typically mutualistic, and results in increased fitness for both partner species, though rhizobia can become parasitic if plants are shaded [15]. A previous study that manipulated the presence of a generalist insect herbivore (*Spodoptera exigua*) on the model legume *Medicago truncatula* in symbiosis with the rhizobium *Sinorhizobium meliloti* showed that herbivory increased nodule numbers and, therefore, increased mutualism benefits to rhizobia [14]. Moreover there was evidence that the strength of this effect was genetically variable among plant populations [14].

Although Heath & Lau [14] found a strong and consistent positive effect of herbivores on nodulation, the mechanism(s) driving this response remained unclear. One potential mechanism might be changes in plant allocation. Herbivory often increases belowground carbon allocation [16], [17], which might indirectly increase nodule numbers. If carbon allocation mediates the herbivory-nodulation response, then one would expect that: 1) any herbivory-nodulation relationship would occur *via* changes in root biomass, and 2) the availability of plant carbon would alter the strength or even direction of these responses. Specifically, shading plants would be expected to lessen any positive relationship between herbivory and nodulation by decreasing the carbon pools available for belowground allocation.

Alternatively, herbivores might increase nodulation via shared signaling networks operating independently from plant carbon status. If shared networks underlie both traits, then variation at these genes would be expected to generate genetic correlations between nodulation and herbivory, regardless of changes in root biomass. For example, jasmonic acid (JA) levels increase after herbivore damage [18], [19], and endogenous JA can increase nodule numbers [20], [21], suggesting shared signaling networks in nodulation and herbivory. In fact, recent experimental work has demonstrated the potential for JA signaling to mediate plant-fungal symbiosis, since exogenously-applied JA can increase mycorrhizal colonization [22], [23]. Importantly, these two alternative mechanisms (carbon allocation and shared signaling networks) are not mutually-exclusive; an organism’s environmentally-influenced condition has the potential to alter the strength or even direction of genetic correlations among traits.

Here we use a quantitative genetics approach to study the context-dependence and genetic architecture of plant interactions with aboveground herbivores and belowground mutualists. Plant genotypes from multiple natural *Medicago truncatula* (barrel medic) [24] populations were grown in the field in either full sun or partial shade. Growth, insect herbivory level, and nodule number were measured in order to: 1) address whether natural levels of herbivory and nodulation are correlated in the field, 2) determine the extent to which changes in root biomass explain herbivory-nodulation correlations, 3) explore the genetic architecture of this suite of plant interaction traits, and 4) ask whether this genetic architecture depends on the light environment.

## Materials and Methods

### Experiment

To assess how the light environment affects the tripartite interactions between plants, their aboveground herbivores, and belowground rhizobium mutualists, we grew 55 plant genotypes in a split-plot design with 30 plots (for 1650 individuals), with either a sun or shade treatment applied at the whole-plot level, and genotypes assigned new randomized locations within each plot. Plant genotypes corresponded to inbred maternal families (hereafter “families”) from eight natural populations in France, in the native range of *M. truncatula*. Each family contained the offspring of a single field-collected maternal individual propagated in the greenhouse for at least one generation in order to minimize maternal environmental effects. Because *M. truncatula* is highly self-fertilizing in nature, individuals from a single maternal family are expected to be nearly genetically uniform [25]; therefore, families are similar to inbred lines. Previous studies of *M. truncatula* have shown among-population and among-family genetic variation for symbiosis with rhizobia [26], [27].

*M. truncatula* seeds were manually scarified and surface-sterilized by dipping briefly in 100% ethanol followed by 7 minutes in commercial bleach, then were glued to individual sterile toothpicks to facilitate planting and seedling identification. Genotypes were randomized into each 2.5 m^2^ experimental block in a freshly tilled agricultural field in Urbana IL, with ∼30 cm spacing between plants. The experiment was watered immediately after planting and approximately thrice per week thereafter, with the exception of rainy days. Blocks in the shade treatment were covered by 2.5 m×2.5 m square of 50% shade cloth suspended 45 cm off the ground by a PVC scaffold. Light levels (PAR) averaged 1277.1±53.5 umol/m^2^s (mean ± SD) in sun, versus 522.3±48.4 umol/m^2^s in shade blocks, as measured using a quantum flux meter (model MQ-200, Apogee Instruments, Logan UT). Shade treatments also decreased soil and leaf temperature (e.g. from an average of 41.8±2.2 C to 32.3±3.0 C for soil and from 32.3±2.1 C to 30.1±1.7 C for leaf), as measured using an infrared thermometer (model 574-CF, Fluke Co., Everett WA) on 12 August, 2010. Probably as a result of the frequent watering necessary to maintain the experiment, soil moisture content did not differ between plots (experiment-wide mean volumetric water content ± stdev = 39.7% ±2.8), as measured using a soil moisture probe (Field Scout TDR 300, Spectrum Technologies, Inc., Plainfield IL) on 17 August, 2010.

Of the initial June 26 planting, 90% of seeds did not emerged, and these individuals were replaced with a second planting on July 16. Because we were interested in how natural levels of herbivory interacted with shade treatments to impact plant growth and nodulation, herbivory was scored as the proportion of damaged leaves (damaged leaves/total leaves) at seven weeks after initial planting. This metric should be correlated with total leaf area removed and moreover should not be biased across treatments. Caterpillars of two lepidopteran species, *Spodoptera ornithogalli* and *Colias eurytheme*, were routinely observed causing the type of herbivore damage typical of experimental plants (P. Knapik, personal observation). Blocks were harvested between September 11 and September 24, 2010. For each plant, the entire root system was dug up, soaked in a bucket of water to loosen and remove all soil, and scored for the total number of nodules present. Aboveground and belowground biomass were then clipped apart, dried, and weighed separately. Because nodules in the field senesce before fruits are ripe, direct estimates of plant reproductive fitness were not feasible.

Germination and survival in the field were low. Planting date (June 26 versus July 16) was not a significant source of variation for any traits and thus was not included in final analyses. Despite the second planting, only 27% of the 1650 planted survived to harvest. To encourage mixed models to converge in order to estimate genetic effects, we included only plant genotypes that were represented by at least two or more individuals per treatment (i.e., at least N = 4 replicates per family) in the analyses. Of the 55 families initially planted, 46 met this criterion; thus although light treatment affected mortality (see Results), differences were not severe enough to lead to heterogeneity in the families represented in the two light treatments. Seedling mortality might have been non-random among families; therefore, the remaining subset might represent a biased sample of the genetic variation, though in what way cannot be known.

### Data Analysis

Except where indicated, analyses were implemented in SAS software (version 9.2, SAS Institute, Cary NC). We used both MANOVA in PROC GLM (all effects treated as fixed) and univariate PROC MIXED to test for the fixed effects of plant population, light treatment, and treatment × population and the random effects of plant maternal family nested within population, treatment × family, and block nested within treatment on each of the four dependent variables. We did not model interactions with block. Pairwise Pearson (phenotypic) correlations were computed among all four traits (nodule number, herbivory, root and shoot biomass) using PROC CORR. Transformations did not dramatically alter the results, but can alter the biological interpretation of data [28]; therefore, we present results of analyses performed on untransformed data.

We used structural equation modeling [29], [30] to tease apart the direct and indirect effects of herbivory on nodule number and how these effects might be altered by light treatment. We created a hypothesized *a priori* model in which herbivory could affect nodule number directly as well as indirectly via root and shoot biomass, using AMOS version 7 [31]. We included a double-headed arrow between root and shoot biomass errors because these two variables are strongly positively correlated with one another, likely due to unmeasured environmental heterogeneity. We also constrained their covariance to be equal between light treatments because the relationship between roots and shoots should be similar in the two environments. We assessed the adequacy of our hypothesized model to describe two types of data (confirmatory analyses sensu [29]). First, we examined the phenotypic data on all individuals, ignoring family structure (hereafter referred to as the ‘phenotypic’ structural equation model or SEM). Second, we examined the population means (hereafter referred to as the ‘population’ SEM; described below). Within each of these two models, we compared how the paths between plant interaction traits differed between the two light treatments (sun and shade) using the critical ratios between the regression coefficients. We did not remove any nonsignificant relationships from the hypothesized models. All data were multivariate normal. The ‘phenotypic’ SEM fit the individual-level data adequately (i.e., was not rejected; χ^2^ = 0.42, d.f. = 1, p = 0.517). Fit indices also suggested that this model fit the data (CFI = 1.0; RMSEA = 0.0 [32].

The family-level (i.e. among-line) genetic variance-covariance (G) matrix describes the broad-sense genetic variances and covariances of traits in each light environment, and thus can illuminate how trait correlations might be expected to influence the outcome of selection on individuals within populations under different light environments. To obtain G matrices, we used repeated measures mixed model analysis of variance implemented in PROC MIXED to model the fixed effects of trait (nodules, herbivory, root biomass, shoot biomass), light treatment (sun or shade), population, population × light treatment, block nested within treatment, and the random effect of plant maternal family nested within population on all phenotypic observations by specifying the “type = UN” solution statement [33], [34]. A grouping term [35] was used in the random statements to estimate separate among-family G matrices for the sun and shade treatments. In order to facilitate model convergence, all traits were first scaled to a mean of 1 by dividing all values by the experiment-wide trait mean (SAS User’s Guide, Version 9.2). All SAS code is available upon request.

We tested for differences in genetic architecture between the two light environments in multiple ways. First, we dropped the grouping term in both random statements and calculated likelihood ratio tests for the null hypothesis of no significant decrease in model fit [35], [36]. Similarly, since we were interested *a priori* in the response of the herbivory-nodulation covariance to light environment, we tested this by comparing the full model (with the grouping term) to one in which this particular covariance was constrained to be equal in the two environments. We tested whether responses to multivariate selection would be expected to change depending on the light environment using the random skewers method of Cheverud [37], but modified for the null hypothesis of matrix equality, implemented in R (version 2.9.0) using a custom program kindly provided by C. Goodnight (raw standardized skewers option, with 1000 bootstrap replicates [38]. Briefly, random skewers multiplies the two matrices by a set of 1000 random vectors and then computes the average correlation of the resulting response vectors, which is then compared to a bootstrap distribution created by randomizing individuals across families for significance testing. Finally, we compared G matrices using a Flury hierarchical analysis implemented in CPCrand using the "jump-up" approach [39]. In the "jump-up" approach, alternative hypotheses about matrix similarity (e.g., whether the matrices share principal components, are proportional to each other or are equal to each other) are each tested against a null model of unrelated matrix structure. CPCrand compares the results of the likelihood-ratio tests between the models to the distribution generated by randomly reassigning families to either sun or shade 1000 times and estimates the probability of obtaining such a test statistic by chance [39].

Because we found significant among-population trait variation, among-population genetic correlations for all traits were also approximated by performing correlations and structural equation modeling (described above) using lsmeans for each population in each treatment (output by the multivariate model described above). The ‘population’ SEM fit the population-level data adequately (χ^2^ = 0.63, d.f. = 1, p = 0.429; CFI = 1.0; RMSEA = 0.0). In contrast to the family-level G matrix, the population-level correlation matrix describes the among-population genetic component of trait variances and covariances [40], and therefore can illuminate how populations from different geographic locations might differ in terms of multivariate trait evolution as a result of spatial genetic structure. It should be noted, however, that a sample size of eight populations is quite small for estimation of among-population variance and covariance; therefore, population-level effects should be interpreted with appropriate caution.

## Results

### Phenotypic Analyses

Univariate ANOVAs and MANOVA indicated that plant population, light treatment, block, and plant maternal family contributed to variation in the dependent variables (Table S1). Mortality was slightly but significantly higher in the sun (74%, versus 70% in the shade; χ^2^_df  = 1_ = 4.21; p = 0.0402). Plants in the shade environment were larger and produced significantly more nodules (mean ± SE; number of nodules: shade = 12.7±1.4; sun = 2.5±1.5), root biomass (shade = 0.23±0.04 g; sun = 0.16±0.04 g) and shoot biomass (shade = 2.0±0.34 g; sun = 1.32±0.35 g) than plants in the sun treatments. In addition to being larger, plants in the shade also experienced less insect damage on a per-leaf basis (proportion of damaged leaves: shade = 0.29±0.02; sun = 0.45±0.02). All traits were phenotypically correlated in this experiment (Table S2). Within both sun and shade environments, nodule number, root biomass, and shoot biomass were positively correlated, while herbivory was negatively correlated with all three remaining variables. Thus smaller and less-nodulated plants also experienced more herbivory in both light environments.

**Figure 1:**
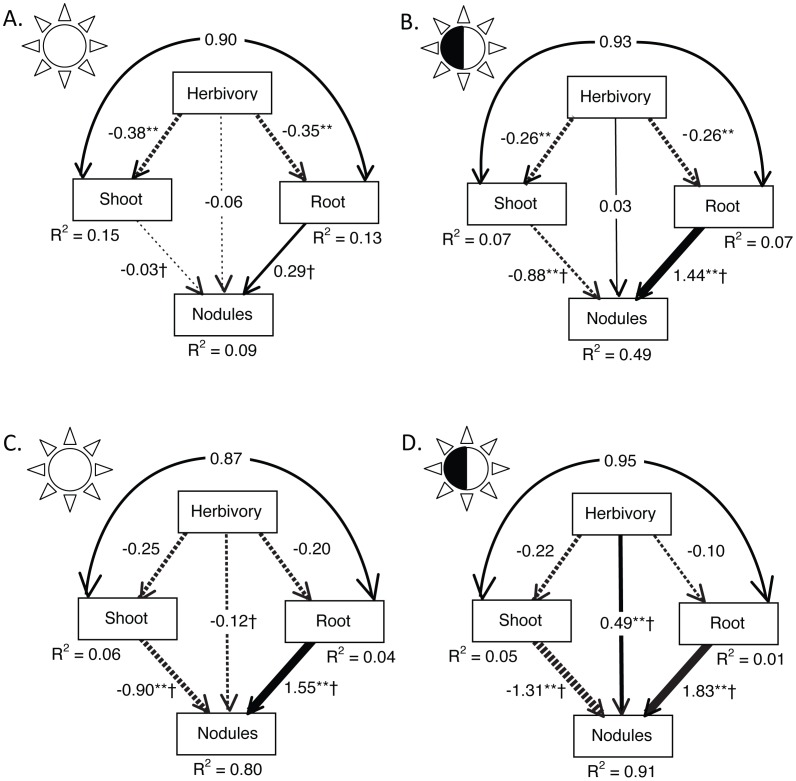
Path diagrams showing the standardized regression weights and the amount of variation in each variable explained by the input arrows (R^2^). Straight arrows reflect causal paths, with the strength of the relationship designated by arrow thickness. Curved arrows designate correlations. Asterisks denote statistically significant paths: *p<0.02, **p<0.001. Paths that are significantly different between sun and shade treatments are indicated with †. Results of the ‘phenotypic’ SEM on all individuals are shown in panels A (sun, N = 682) and B (shade, N = 681); those of the ‘population’ SEM on population means are shown in panels C (sun, N = 8) and D (shade, N = 8).

We used ANOVA paired with structural equation modeling (SEM) to tease apart the effects of these correlated traits on nodule number. Most importantly, the non-significant direct effects of herbivory on nodulation in both sun and shade (Table 1; Figure 1A,B; see Figure S1 for unstandardized regression weights) indicated that the overall negative phenotypic correlations between herbivory and nodulation were mediated entirely through plant biomass. Not surprisingly, both root and shoot biomass were negatively influenced by herbivory in both environments, and the directions of downstream effects of biomass on nodule number were consistent between sun and shade environments (Figure 1A,B). The standardized total effect (indirect and direct) of herbivory on nodule number was −0.153 in the sun and −0.116 in the shade. Nevertheless significant interactions of root and shoot biomass with light treatment in the ANOVA (Table 1), as well as significant differences in the biomass-nodule number paths in the sun versus shade SEM (comparing paths in sun and shade: shoot to nodules, z-value = −5.837, P<0.0001; root to nodules, z-value = 9.545, P<0.0001; Figure 1A,B), indicated that the slopes of the root-nodule number and shoot-nodule number relationships differed between light environments. In particular, neither shoot nor root biomass significantly affected nodule number in the sun treatment (Figure 1A), while both significantly affected nodule number in the shade treatment (Figure 1B).

**Table 1:** ANOVA on phenotypic data for all individuals: the effects of light treatment (sun or shade), herbivory, shoot biomass, root biomass, and interactions with light treatment on nodule number.

	Nodule number		
*Source*	*Mean square*	*F_1,404_*	*p*
Light Treatment	12.30	0.17	0.6787
Herbivory	1.70	0.02	0.8775
**Root Biomass**	**7813.56**	**109.20**	**<0.0001**
**Shoot Biomass**	**1919.59**	**26.83**	**<0.0001**
Light Treatment × Herbivory	57.34	0.80	0.3712
**Light Treatment** × **Root Biomass**	**6607.60**	**92.34**	**<0.0001**
**Light Treatment** × **Shoot Biomass**	**2303.83**	**32.20**	**<0.0001**

### Genetic Analyses

In order to assess the genetic architecture underlying this suite of correlated plant traits in the field, we first estimated G matrices representing the among-family (inbred line) genetic component (i.e. broad-sense genetic variation; Table 2).

**Table 2:** Broad-sense genetic variances (diagonal, in italics) and covariances (off-diagonal) for nodule number, herbivory, root biomass, and shoot biomass of field-grown *M. truncatula* plants in the two light treatments (sun or shade).

	Nodule number		Herbivory		Root biomass		Shoot biomass	
Nodule number	*0.1195(3%)*	*0.0151(5%)*	0.0419		−0.0787		−0.0494	
Herbivory	0.0448		*0(0%)*	*0(0%)*	0.0021		−0.0063	
Root biomass	0.0303		0.0192		*0.0565(100%)*	*0(0%)*	0.0237	
Shoot biomass	−0.0003		0.0174		0.0396		*0.0026(3%)*	*0.0871(22%)*

Coefficients of variation for each trait are shown in parentheses. Shade variances and covariances are below the diagonal, and sun variances and covariances are above the diagonal.

We used multiple approaches to test whether the genetic architecture of these traits differed significantly between light environments, with differing results. Model fit was improved when G matrices were estimated separately for each light treatment (χ^2^ = 252.1, df = 20, p<0.0001). Nevertheless, the main covariance of interest, that between herbivory and nodulation, was equal in the two light environments; specifically, a model in which sun and shade covariances were estimated separately was not significantly more likely than a model in which they were constrained to be equal (χ^2^ = 0.3, df = 1, p = 0.2919). Responses to multivariate selection in the sun versus shade environment were not predicted to differ significantly using the null hypothesis-corrected random skewers method (r = 0.56, p = 0.86). Finally Flury hierarchical analysis indicated that, although the matrices were not equal or even proportional (likelihood ratio = 3.3991, p<0.0001), their eigenstructure was similar (i.e., we could not reject the model of common principle components, likelihood ratio = 1.9645, p = 0.0606). In sum, these results provide little evidence that responses to selection on these interaction traits in *M. truncatula* would differ drastically between light environments.

More genetic variation for nodulation was found among populations than among plant families in this experiment (Table S1). Both ANOVA and SEM approaches to address this component of variance suggested that among-population differences in nodulation were driven by all three remaining traits (herbivory, root biomass, and shoot biomass; Table 3; Figure 1C,D). The ‘population’ SEM revealed no evidence for among-population correlations between herbivory and shoot or root biomass (Figure 1C,D). Like the ‘phenotypic’ SEM (Figure 1A,B), root and shoot biomass were positively and negatively (respectively) correlated with nodulation, and the strength of both relationships increased significantly in the shade (comparing paths in sun and shade: root to nodules, z-value = 3.205, p = 0.001; shoot to nodules, z-value = −2.584, p = 0.010; Figure 1C,D). In contrast to the phenotypic analyses, however, but consistent with the family-level genetic covariances (Table 2), herbivory had a positive and direct effect on nodule number (Table 3), but only in the shade (comparing paths in sun and shade: herbivory to nodules, z-value  = 3.991, p<0.0001; Figure 1D). An important consequence of the light environment is that it altered the direction and magnitude of the standardized total effect (sum of indirect and direct) of herbivory on nodule number, from −0.208 in the sun to 0.596 in the shade. In sum, to the extent that these eight populations represent the among-population component of genetic variance, these results are consistent with a shared, but environmentally-dependent, genetic component underlying nodulation and herbivory.

**Table 3:** ANOVA on population means: the effects of light treatment (sun or shade), herbivory, shoot biomass, root biomass, and interactions with light treatment on nodule number.

	Nodule number		
*Source*	*Mean square*	*F_1,8_*	*p*
Light Treatment	0.11	2.5	0.1521
**Herbivory**	**0.47**	**10.7**	**0.0114**
**Shoot Biomass**	**1.20**	**27.2**	**0.0008**
**Root Biomass**	**0.55**	**12.4**	**0.0078**
**Light Treatment** × **Herbivory**	**0.55**	**12.5**	**0.0077**
**Light Treatment × Root Biomass**	**0.31**	**7.0**	**0.0299**
Light Treatment **×** Shoot Biomass	0.21	4.7	0.0621

## Discussion

Plants participate simultaneously in numerous species interactions, and these multi-player interactions have the potential to alter both ecological and evolutionary outcomes in natural populations. We used a quantitative genetics approach and manipulated the light environment in the field to explore environmental and genetic influences on plant traits that mediate interactions with other species - both the aboveground interactions with herbivore antagonists and the belowground interactions with rhizobium mutualists. We found that shifts in plant biomass allocation can affect indirect interactions between insect herbivores and rhizobia. However positive among-family and among-population genetic covariances between herbivory and nodulation do suggest that shared genes might underlie plant responses to both types of interactors. Finally, there was evidence that the environment might alter the genetic architecture of these plant interaction traits. We highlight potential ecological and evolutionary implications of these results below.

### Plant Carbon Allocation Mediates Tripartite Interactions

We predicted that, if plant carbon allocation mediates the effects of herbivores on nodulation, then any herbivory-nodulation relationship would be explained by changes in root and/or shoot biomass and would be affected by carbon availability (i.e., shade). Although our results suggest a role for biomass allocation in mediating these interactions, it is not in the expected direction. More nodulated plants in this experiment had lower levels of herbivory (both within and among light treatments), and this phenotypic relationship between herbivory and nodulation was entirely mediated via changes in plant biomass allocation. Other authors have also found that larger plants experience less herbivory, possibly because plants in more favorable conditions have more resources to allocate to both growth and defense [41]. Given the apparent poor condition of plants in the current experiment, this explanation seems feasible: whether it was the imposed difference between sun and shade treatments or random micro-environmental variation, plants in more favorable conditions were larger, had more nodules, and were more resistant to insect herbivores in both light environments. Very generally, this result is consistent with models of mutualism [42], [43] that predict positive feedback between the condition or fitness of one partner (here, the plant) and the population size of its partner species (here, the rhizobium).

### Plant Genotype Mediates Tripartite Interactions

In contrast to the negative phenotypic correlation between herbivory and nodule number we observed here, a previous manipulative study in this system found that herbivory increased nodule numbers [14], and suggested that a shared genetic basis for herbivore and rhizobium responses by *M. truncatula* could generate such a pattern. Here we predicted that, if shared signaling networks underlie both responses, then positive genetic correlations should exist between herbivory and nodulation. The positive genetic covariances/correlations between nodulation and herbivory found here at both the among-family and among-population level – even after controlling for changes in plant size – are indeed consistent with a shared genetic basis for plant responses to herbivores and rhizobia. Importantly, because neither nodulation nor herbivory was manipulated in this experiment, these results could be driven by either interactor.

It is important to note that genetic covariance at any geographic scale can be generated by three distinct causes: pleiotropy resulting from shared genetic mechanisms, physical linkage, or linkage disequilibrium resulting from historical evolutionary processes [44], [45]. Thus the extent to which nodulation-herbivory covariance in this experiment is driven by overlapping response pathways remains a mystery, though current molecular techniques have the potential to unravel the molecular basis of context-dependent trait correlations such as these [46], [47]. The role of phytohormones such as JA in regulating nodulation is still being elucidated [21], [48], and multiple genetic pathways might underlie shared aboveground and belowground responses by plants. Nevertheless given JA’s role in herbivory [18], [19] and nodulation [21], as well as recent empirical evidence that plant JA might mediate tripartite interactions between pollinators, plants, and arbuscular mycorrhizal fungi [23], the overlapping role of JA in herbivore antagonism and rhizobial mutualism is likely a profitable avenue for future work.

Genetic variance in nodulation was found among populations, in addition to among families within populations, in this experiment. Previous studies in this and other systems have found population-level genetic variation in either nodulation or herbivory [27], [49], though studies of *M. truncatula* have typically found that most quantitative genetic variation exists within populations [14], [27], [50]. Our results indicated that, among the eight populations studied here, those populations that produced more nodules tended to be more susceptible to insect herbivores. This stands in contrast to the phenotypic pattern, possibly because these traits are simultaneously linked by the environment and underlying genes. To the extent that these correlations do reflect shared plant genetic mechanisms (JA signaling or otherwise, which remain to be tested), results like ours suggest that the evolution of nodulation (and plant-rhizobium coevolution) would not proceed independently from the evolution of resistance (and plant-herbivore coevolution). Such findings strengthen the argument for including multi-player interactions, and community genetics in general, in ecological and evolutionary studies [51].

Population-level genetic covariance, such as that found between herbivory and nodulation in the current experiment, has long been a subject of interest and debate in evolutionary biology. Analogous to the effect of correlated traits on population evolution [44], traits that are correlated among populations have the potential to alter population-level (group) evolution by constraining the directions in multivariate trait space that are available to evolution [40], [52]. Population-level correlations may also reveal the historical action of geographically-variable selection for adaptive trait complexes [53]; however, population-level trait covariance can also result from neutral evolutionary processes like drift and gene flow [54]. Thus it is not possible to know the extent to which population-level correlations between nodulation and herbivory in this experiment are driven by shared genetic mechanisms, versus historical evolutionary processes. The evolutionary dynamics of metacommunities, i.e. multiple interacting populations coevolving in a spatial context, are underexplored [4]. We propose that trait correlations among characters that mediate key interactions in natural communities, such as those explored here, might play important roles in the evolutionary trajectory of community evolution at multiple spatial scales. Our experiment provides a foray into among-population variance in tripartite interactions and suggests testable hypotheses. Appropriate genetic designs that maximize population-level sampling, while difficult experimentally, will be critical for rigorous estimates of these variance components in the future.

### The Light Environment Mediates Tripartite Interactions

Because herbivores and rhizobia both utilize plant carbon, we predicted that carbon limitation imposed by a shade treatment might generate negative correlations (phenotypic or genetic) between herbivory and nodulation. We found little evidence supporting this prediction. Negative phenotypic correlations between herbivory and nodulation in both environments were mediated entirely via changes in root and shoot biomass, probably reflecting a more general plant tradeoff between aboveground and belowground allocation [55]. Indeed the only significant direct relationship between herbivory and nodulation was a positive among-population genetic correlation within the shade treatment. More generally, the major axes of trait variation in this experiment were largely consistent across light environments, providing weak evidence that multivariate responses to selection would differ between light treatments. To the extent that interaction trait covariances/correlations (such as the ones studied here) do differ among environments, the strength and importance of the resulting indirect effects and metacommunity evolutionary processes would be expected to vary with the environmental context.

## Supporting Information

Figure S1 **Path diagrams showing the unstandardized regression weights and the amount of variation in each variable explained by the input arrows (R^2^).** Straight arrows reflect causal paths, with the strength of the relationship designated by arrow thickness. Curved arrows designate correlations. Results of the ‘phenotypic’ SEM on all individuals are shown in panels A (sun, N = 682) and B (shade, N = 681); those of the ‘population’ SEM on population means are shown in panels C (sun, N = 8) and D (shade, N = 8).
(TIF)

Table S1 MANOVA and univariate mixed model ANOVA (using REML) results for the effects of light treatment (sun or shade), plant population, maternal family and block on nodulation, insect herbivory, and root and shoot biomass of field-grown *M. truncatula*. For MANOVA, Wilks Lambda is shown; in univariate models, F-statistics are shown for fixed effects, and χ^2^ statistics (df = 1) are shown for random effects, as well as percent variance explained (PVE).
(DOCX)

Table S2 Phenotypic correlations (N) between dependent variables in the two light treatments (sun or shade). Trait correlations in shade are in grey below the diagonal; trait correlations in sun are in white above the diagonal. ^*^p<0.1; ^**^p<0.01;^***^p<0.001;^****^p<0.0001
(DOCX)
