# Supplementary figures and images for: Coevolutionary Constraints? The Environment Alters Tripartite Interaction Traits in a Legume
Source: PLoS One. 2012 Jul 30;7(7):e41567. doi: 10.1371/journal.pone.0041567 (PMC3408487; doi:10.1371/journal.pone.0041567)

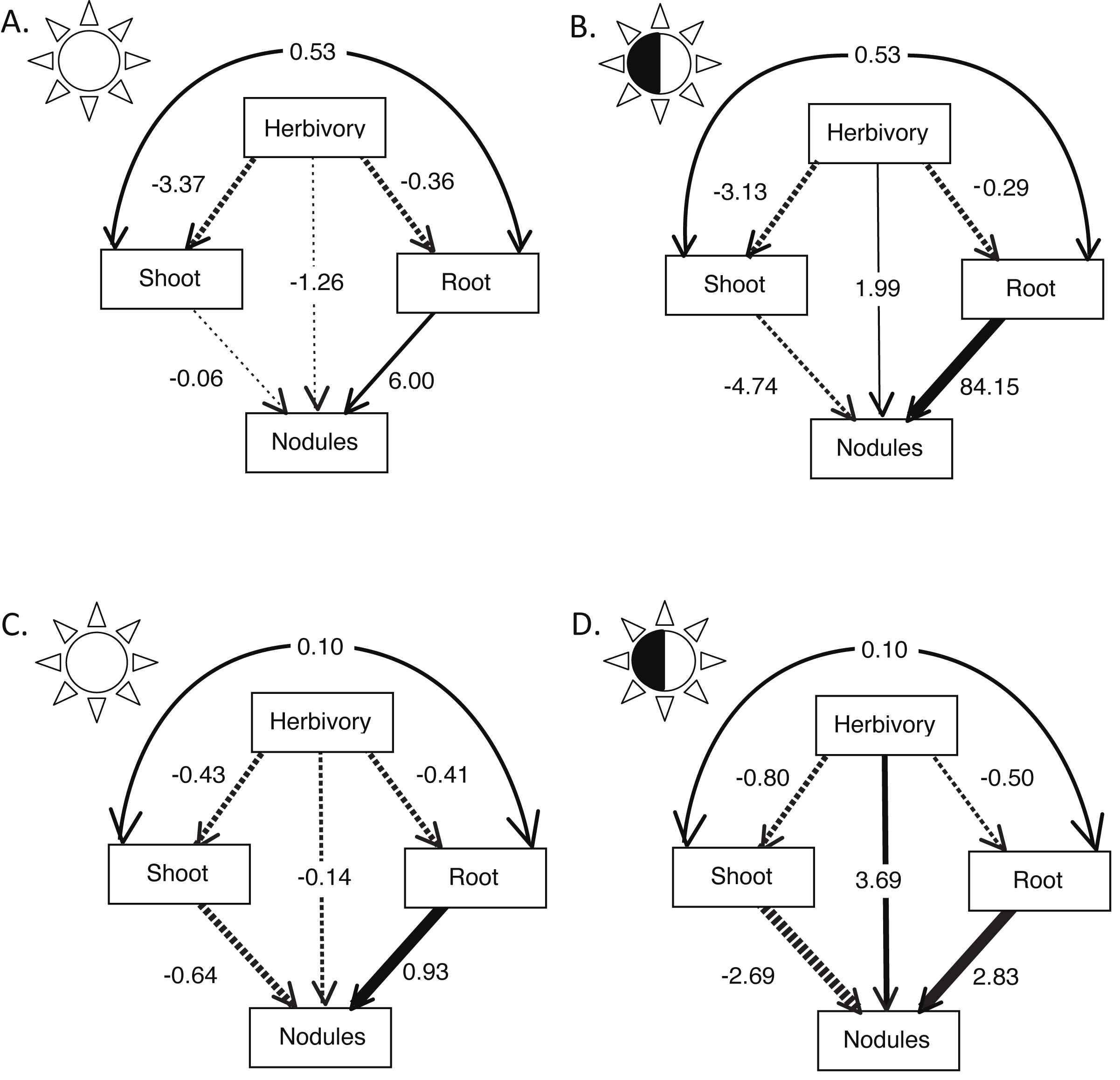

Supplement: Figure S1 — Path diagrams showing the unstandardized regression weights and the amount of variation in each variable explained by the input arrows (R2). Straight arrows reflect causal paths, with the strength of the relationship designated by arrow thickness. Curved arrows designate correlations. Results of the ‘phenotypic’ SEM on all individuals are shown in panels A (sun, N = 682) and B (shade, N = 681); those of the ‘population’ SEM on population means are shown in panels C (sun, N = 8) and D (shade, N = 8). (TIF) [file pone.0041567.s001.tif]
