# Supplementary material for: Coevolutionary Constraints? The Environment Alters Tripartite Interaction Traits in a Legume
Source: PLoS One. 2012 Jul 30;7(7):e41567. doi: 10.1371/journal.pone.0041567 (PMC3408487; doi:10.1371/journal.pone.0041567)
Supplement: Table S1 — MANOVA and univariate mixed model ANOVA (using REML) results for the effects of light treatment (sun or shade), plant population, maternal family and block on nodulation, insect herbivory, and root and shoot biomass of field-grown M. truncatula. For MANOVA, Wilks Lambda is shown; in univariate models, F-statistics are shown for fixed effects, and χ2 statistics (df = 1) are shown for random effects, as well as percent variance explained (PVE). (DOCX) [file pone.0041567.s002.docx]

**Supplementary Table 1** MANOVA and univariate mixed model ANOVA (using REML) results for the effects of light treatment (sun or shade), plant population, maternal family and block on nodulation, insect herbivory, and root and shoot biomass of field-grown *M. truncatula*. For MANOVA, Wilks Lambda is shown; in univariate models, F-statistics are shown for fixed effects, and χ^2^ statistics (df = 1) are shown for random effects, as well as percent variance explained (PVE).

|  | MANOVA | | Nodule number | | | Herbivory | | | Root biomass | | | Shoot biomass | | |
| --- | --- | --- | --- | --- | --- | --- | --- | --- | --- | --- | --- | --- | --- | --- |
| Source |  |  |  | *P* | *PVE* |  | *P* | *PVE* |  | *P* | *PVE* |  | *P* | *PVE* |
| Population | **F_28,127.6_= 1.94** | **0.0072** | **F_7,37_ = 3.70** | **0.0040** | **8.1** | F_7,37_ = 0.73 | 0.6441 | 0 | **F_7,37_= 3.23** | **0.0090** | **17.0** | F_7,37_= 1.53 | 0.1861 | 4.8 |
| Light treatment | **F_4,291_ = 22.32** | **<0.0001** | **F_1,28_ = 25.64** | **<0.0001** | **57.8** | **F_1,28_ = 22.28** | **<0.0001** | **78.0** | F_1,28_ = 2.29 | 0.1411 | 3.3 | F_1,28_ = 2.12 | 0.1562 | 2.7 |
| Treatment × Population | F_28,127.6_=  1.19 | 0.2499 | F_7,37_ = 1.85 | 0.1064 | 4.5 | F_7,37_ = 1.25 | 0.3010 | 0 | F_7,37_ = 1.43 | 0.2225 | 4.3 | F_7,37_ = 1.12 | 0.3690 | 2.4 |
| Family (Pop) | **F_152,1162.2_ = 1.27** | **0.0195** | χ^2^ = 1.4 | 0.1184 | 3.5 | χ^2^ = 0 | 1 | 0 | **χ^2^ = 3.0** | **0.0416** | **15.3** | χ^2^ = 2.5 | 0.0569 | 14.6 |
| Treatment × Family (Pop) | F_144,1161.7_ = 0.89 | 0.8187 | χ^2^ = 0 | 1 | 0 | χ^2^ = 0 | 1 | 0 | χ^2^ = 0 | 1 | 0 | χ^2^ = 0 | 1 | 0.0 |
| Block (Treatment) | **F_112,1158.3_ = 2.61** | **<0.0001** | **χ^2^ = 41.9** | **<0.0001** | **26.1** | **χ^2^ = 10** | **0.0007** | **22.0** | **χ^2^ = 39.9** | **<0.0001** | **60.1** | **χ^2^ = 40.4** | **<0.0001** | **75.5** |
