# Supplementary material for: Coevolutionary Constraints? The Environment Alters Tripartite Interaction Traits in a Legume
Source: PLoS One. 2012 Jul 30;7(7):e41567. doi: 10.1371/journal.pone.0041567 (PMC3408487; doi:10.1371/journal.pone.0041567)
Supplement: Table S2 — Phenotypic correlations (N) between dependent variables in the two light treatments (sun or shade). Trait correlations in shade are in grey below the diagonal; trait correlations in sun are in white above the diagonal. *p<0.1; **p<0.01;***p<0.001;****p<0.0001 (DOCX) [file pone.0041567.s003.docx]

**Supplementary Table 2** Phenotypic correlations (N) between dependent variables in the two light treatments (sun or shade). Trait correlations in shade are below the diagonal; trait correlations in sun are above the diagonal. ^*^p< 0.1; ^**^p< 0.01;^***^p< 0.001;^****^p< 0.0001

|  | Nodules | Herbivory | Root biomass | Shoot biomass |
| --- | --- | --- | --- | --- |
| Nodules |  | -0.1985^*^ | 0.3499^****^ | 0.2844^****^ |
| Herbivory | -0.1348^*^ |  | -0.3547^****^ | -0.3542^****^ |
| Root biomass | 0.6186^****^ | -0.2715^****^ |  | 0.8832^****^ |
| Shoot biomass | 0.4325^****^ | -0.2830^****^ | 0.9033^****^ |  |
